# Bayesian-Inference Embedded Spline-Kerneled Chirplet Transform for Spectrum-Aware Motion Magnification

**DOI:** 10.3390/s22072794

**Published:** 2022-04-06

**Authors:** Enjian Cai, Dongsheng Li, Jianyuan Lin, Hongnan Li

**Affiliations:** 1 Department of Civil Engineering, Tsinghua University, Beijing 100084, China; cej21@mails.tsinghua.edu.cn; 2 Department of Civil and Environmental Engineering, Guangdong Engineering Center for Structure Safety and Health Monitoring, Shantou University, Shantou 515063, China; 19jylin@stu.edu.cn; 3 State Key Laboratory of Coastal & Offshore Engineering, Dalian University of Technology, Dalian 116023, China; hnli@dlut.edu.cn

**Keywords:** motion magnification, time-spectrum analysis, statistical inference, computer vision

## Abstract

The ability to discern subtle image changes over time is useful in applications such as product quality control, civil engineering structure evaluation, medical video analysis, music entertainment, and so on. However, tiny yet useful variations are often combined with large motions, which severely distorts current video amplification methods bounded by external constraints. This paper presents a novel use of spectra to make motion magnification robust to large movements. By exploiting spectra, artificial limitations and the magnification of small motions are avoided at similar frequency levels while ignoring large ones at distinct spectral pixels. To achieve this, this paper constructs spline-kerneled chirplet transform (SCT) into an empirical Bayesian paradigm that applies to the entire time series, giving powerful spectral resolution and robust performance to noise in nonstationary nonlinear signal analysis. The important advance reported is Bayesian-rule embedded SCT (BE-SCT); two numerical experiments show its superiority over current approaches. For applying to spectrum-aware motion magnification, an elaborate analytical framework is established that captures global motion, and use of the proposed BE-SCT for dynamic filtering enables a frequency-based motion isolation. Our approach is demonstrated on real-world and synthetic videos. This approach shows superior qualitative and quantitative results with less visual artifacts and more local details over the state-of-the-art methods.

## 1. Introduction

Video motion magnification techniques have opened up a wealth of important applications. Examples include detecting a heartbeat vibration from tiny head motions [1] or blood flow [2], magnifying muscle tremors [3,4] to give an accurate clinical judgement, reconstructing speech information from small visual variations [5], evaluating material properties by the way it moves [6], estimating damage information of a building structure by measuring small vibrations in video [7,8,9], and lip reading [10]. However, some essential properties of dynamic objects become evident only when they move. For example, the muscles of an athlete when doing sports, the mechanical properties of a drone in flight, or the tremors of a Parkinson patient during walking. To open up new applications, this paper proposes to amplify variations at dynamic spectrum ranges in the entire time series, which makes motion amplification robust to occlusions and large motions.

Current video magnification techniques are classified into two categories: Lagrangian and Eulerian perspective. For Lagrangian approaches [11], tiny visual variations can be magnified by explicitly estimating feature points with optical flow, but corruptions affect amplification quality easily, since the local motion is represented by a single feature pixel point. The Eulerian techniques, on the other hand, do not estimate motions explicitly. Instead, they decompose video frames into representations to manipulate the local motions, thus handling noise at deeper levels. In [2], an input video frame sequence is first decomposed into a multiscale stack (Laplacian or Gaussian pyramids); then, subtle changes are temporally filtered to find the variations to be amplified. When scaled and added back to the input images, a magnified output is rendered. With the complex-steerable pyramid [12,13,14], an input video frame sequence can be decomposed into a multi-orientation, multiscale stack [15]. Moreover, the use of phase-based motion processing has been considered not only in the context of motion magnification but also for many motion-related cases. In [5], phase decomposition is employed for extracting sound information from high-speed cameras, whereas in [16], the video phase information is applied to predict object material properties, and in [17], phase aids in estimating measurements of structural vibrations. In the context of motion amplification, the successful work in [15] extracts phase information through complex steerable filters and then magnifies it, the phase-based technique has better noise handling characteristics and supports larger amplification factors. In [18], a significant speedup without perceptual decrease in quality has been obtained; this work approximates the complex pyramid with the Riesz pyramid. While extremely successful for clean video signals, all these approaches assume that the objects of interest have very small motion—no large object motion or camera motion existing. Such large motions when processed result in large artifacts such as haloes or ripples, and our technique is specially designed to deal with these cases.

To deal with large motions, a layer-based video magnification approach was proposed in [19], with some help of a manually drawn mask by the user, outlining regions whose pixels are specified to be tracked and then magnified and yielding good magnification results. Whereas a mask indicates which pixels should be used, motion filter effects on the border of the mask cannot be ignored, leading to a certain spatial extent and eventually leaking across the mask edge. On the other hand, manual selection is time consuming and error prone; the selected region tracking is sensitive to occlusions and 3D object rotations. Furthermore, the alignment is based on a homography, which may generate wrong information for non-planar objects and a non-static camera. By using depth cameras and bilateral filters, the recent work in [20] proposed an alternative approach, making it possible for the amplification processing to be applied on pixels located at the same depth. In a sense, depth-aware motion processing extends the layer-based approach in replacing the manual selection mask by a weighted mask obtained from depth ranges, which avoids manual annotation to some extent. In addition, it also prevents the leaking problem in [19] by ignoring motion effects from different depth layers. However, this technique cannot cope with any moving objects; more importantly, the lack of depth knowledge will introduce inaccurate manual operations in processing. Based on the assumption that the large motion is typically linear at the scale of the small variations, the innovative processing framework [21] was proposed to magnify small deviations of linear motion by linking the response of a second-order Gaussian derivative to spatial acceleration. This work achieves impressive results for motion magnification; however, the downside is the inability to cope with nonlinear large motion. Inspired by the above approaches, this essay exploits time-frequency characteristics to automatically define the mask. In addition, based on the observation in [21,22], the significant differences are found in the frequency domain between these two kinds of variations, making our technique in principle suitable for large motion isolation.

Several techniques are available for time-frequency analysis, having played important roles in analyzing nonstationary signals. Among a number of analysis methods, short-time Fourier transform (STFT) [23,24,25], Wigner–Ville distribution (WVD) [26,27], wavelet transform (WT) [28,29], and Hilbert–Huang transform (HHT) [30,31] have been widely applied. For the STFT approach, since the STFT is based on traditional Fourier transform, the signal is assumed to be piecewise stationary at the scale of the window width, so showing weakness in accurate estimation for time-varying frequency. Whereas extremely successful for presenting excellent time-frequency representation for signals in terms of energy concentration, the WVD’s bilinear structure creates the redundant cross terms that cannot track the true time-frequency structure of the signal well, leading to the inaccurate estimation of instantaneous frequency. As another form of STFT method with an adjustable window size, the WT uses a large window for low-frequency components and a small window for high-frequency components, so it cannot achieve an accurate estimation for time-varying frequency as well [25]. Via applying the combination of empirical mode decomposition (EMD) and Hilbert spectral analysis, HHT offers a powerful way for nonstationary nonlinear signal analysis, which makes the instantaneous frequency meaningful. The need for spurious harmonics to represent nonlinear and nonstationary signals is also eliminated. However, the shortcomings of HHT include envelope fitting, mode mixing, end effects of EMD, and no uniform criterion for sifting stop, which may yield misleading results in nonstationary nonlinear signal analysis. By introducing an extra chirp kernel, which is characterized by the chirping rate parameter, the time-frequency atoms of the Chirplet transform (CT) can be sheared and shifted to match the signal in the time-frequency plane, thus showing superiority to WT and other time-frequency analysis approaches in analyzing nonstationary signals. Nonetheless, due to the inability of the chirp kernel to suit nonlinear-frequency-modulated (NLFM) signals, the CT fails the identification and extraction of the nonlinear frequency of the NLFM signal. By replacing the kernel of a frequency-shift operator and a frequency-rotate operator with spline kernel function [32], the spline-kerneled chirplet transform (SCT) extends the capability of the CT and is able to produce a time-frequency representation with an excellent energy concentration for signals with nonlinearly time-varying instantaneous frequency, such that the instantaneous frequency of the NLFM signal can be accurately estimated. This paper begins with the SCT due to its superiority over other time-frequency analysis techniques. However, a critical shortcoming remains in current time-frequency methods, including SCT. These widely used techniques lack a statistical inference framework applicable to the entire time series [33,34,35,36,37,38,39]; their spectrum estimates on adjacent intervals cannot be formally related. Therefore, this paper adapts SCT by constructing a Bayesian statistical inference framework so it can be applied to wider practical projects.

In the following sections, the current parameterized time-frequency analysis techniques will be first discussed; then, we offer a statistical inference framework on how to model nonstationary time series on nonoverlapping intervals. The improved technique is experimentally evaluated by comparing against the state-of-the-art approaches in two numerical examples, and application of the proposed algorithm for video magnification in the presence of large motion is also shown to yield a superior performance over the existing amplification method.

## 2. Proposed Be-Sct

In this section, based on the traditional SCT (readers interested in this theory, please refer to [34,40,41,42]), the nonstationary time signal is modeled as a series of second-order stationary Gaussian processes defined on nonoverlapping spline function intervals. After SCT processing, a frequency domain random-walk model is utilized to relate the spectral representations of the Gaussian processes. The proposed algorithm efficiently calculates spectral updates by parallel complex Kalman filters; moreover, an expectation–maximization (EM) algorithm is utilized to estimate static and dynamic model parameters. The estimate is empirical Bayes, because it is computed conditional on the maximum likelihood parameter estimates.

SCT entails estimating the frequency content as a spline function of time for the nonstationary signal, and it is carried out by repeating spectrum estimation time intervals. Nevertheless, spectrum estimates on adjacent intervals are not regularly related. In contrast, current time-frequency methods are computationally intensive, achieve their high performance in signal-to-noise problems, and up to now have had limited application in practical time series analyses. Despite their usefulness for studying important problems, a critical shortcoming remains in current time-frequency methods including SCT: none of them offers an efficient statistical inference framework appropriate for the entire time series.

State-space modeling is an established, flexible inference framework for analyzing systems with properties that change over time [41,42]. In addition, this paradigm has been widely applied for the analysis of nonstationary time series with harmonic regression models [43], parametric time series models [44,45], and nonparametric time series models based on batch processing [46,47]. Therefore, on the basis of SCT, a plausible approach to analyze nonstationary and oscillatory time series can be proposed. By providing a flexible time-domain decomposition of the time series and a broadly applicable, empirical Bayes’ framework for statistical inference, a comprehensive analysis framework for time-varying spectral analysis of nonstationary nonlinear time series can be achieved. The crucial advance reported is specially constructed Bayesian-rule embedded SCT (BE-SCT).

### 2.1. Theory

In the time-frequency model of BE-SCT, a nonstationary nonlinear time series observed can be defined as:

(1) $$y_{t}=x_{t}+\varepsilon_{t}$$

where $x_{t}$ is a second-order, zero mean, locally stationary Gaussian process, and $\varepsilon_{t}$ is a zero mean, independent Gaussian noise with common variance $\sigma_{\varepsilon }^{2}$. A common approach in the analysis of nonstationary time series is to assume a minimum interval length on which the data are stationary. The stationary intervals are indexed as: i=1,2,…,I, where *I* defines the number of distinct, nonoverlapping stationary intervals in $x_{t}$.

Based on the spectral representation theorem [48], the form of the observation model on stationary interval *i* is defined as:

(2) $$\begin{array}{cc} Y_{i} & =X_{i}+\varepsilon_{i}\\ \; & =\Delta Z_{i}+\varepsilon_{i} \end{array}$$

where $\varepsilon_{i}$ denotes an independent, zero mean Gaussian noise with common variance $\sigma_{\varepsilon }^{2}$.

To relate the data on adjacent intervals, the Gaussian increment differences are assumed to be linked by the random walk model.

(3) $$\Delta Z_{i}=\Delta Z_{i-1}+v_{i}$$

where $v_{i}$ is assumed to be an independent, zero mean complex Gaussian process. In Equation (3), a stochastic continuity constraint is defined on the nonstationary time series in the frequency domain.

Followed by applying SCT, to represent the observation model Equation (2) in the frequency domain, the SCT operator is introduced, thus yielding the equation:

(4) $$\begin{array}{c} Y_{i,s}^{\left(SCT\right)}=\Delta Z_{i,s}^{\left(SCT\right)}+\varepsilon_{i,s}^{\left(SCT\right)}\\ \Delta Z_{i,s}^{\left(SCT\right)}=\Delta Z_{i-1,s}^{\left(SCT\right)}+v_{i,s}^{\left(SCT\right)} \end{array}$$

where *s* denotes the number of observations per stationary interval in the time-frequency plane, $Y_{i,s}^{\left(SCT\right)}=Y_{i}*{SCT}_{s}$, $\varepsilon_{i,s}^{\left(SCT\right)}=\varepsilon_{i}*{SCT}_{s}$ is a zero mean, complex Gaussian vector, and $v_{i,s}^{\left(SCT\right)}=v_{i}*{SCT}_{s}$ is also a zero mean, independent complex Gaussian vector. For ease of reading, the superscript “${}^{\left(SCT\right)}$” is omitted in the following derivations.

### 2.2. Algorithm

According to the linear complex Gaussian form of Equation (4), the sequence of increment differences [44] can be computed by a Kalman filter algorithm. The Gaussian increment difference estimates are assumed to have been computed on interval $\left(i-1\right)$, then for line *s*, a 1D complex Kalman filter algorithm for estimating $\Delta Z_{i,s}\left(\omega_{s}\right)$ on interval *i* can be obtained:

(5) $$\begin{array}{cc} \Delta Z_{i|i-1,s} & \left(\omega_{s}\right)=\Delta Z_{i-1|i-1,s}\left(\omega_{S}\right)\\ \sigma_{i|i-1,s}^{2} & =\sigma_{i-1|i-1,s}^{2}+\sigma_{v,S}^{2}\\ \Delta Z_{i|i,s}\left(\omega_{s}\right)=\Delta Z_{i|i-1,s} & \left(\omega_{s}\right)+C_{i,s}\left(Y_{i,S}-\Delta Z_{i|i-1,S}\left(\omega_{s}\right)\right)\\ \sigma_{i|i,s}^{2} & =\left(1-C_{i,s}\right)\sigma_{i|i-1,s}^{2} \end{array}$$

The Kalman gain for i=1,…,I and s=1,…,S can be computed as:

(6) $$C_{i,s}={\left(\sigma_{\varepsilon }^{2}+\sigma_{i|i-1,s}^{2}\right)}^{-1}\sigma_{i|i-1,s}^{2}$$

The definition i|u is the estimation on the stationary interval *i* based on all of the signal data observed through stationary interval *u*.

To efficiently analyze the functions of the increment differences at any time, the joint distribution of the increment differences in the time series can be computed using the fixed interval smoothing algorithm, which is defined as:

(7) $$\begin{array}{cc} \Delta Z_{i|I,s}\left(\omega_{s}\right)=\Delta Z_{i|i,s}\left(\omega_{s}\right)\; & +A_{i,s}\left(\Delta Z_{i+1|I,s}\left(\omega_{s}\right)-\Delta Z_{i+1|i,s}\left(\omega_{s}\right)\right)\\ \sigma_{i|I,S}^{2}=\sigma_{i|i,S}^{2} & +A_{i,s}^{2}\left(\sigma_{i+1|I,S}^{2}-\sigma_{i+1|i,S}^{2}\right)\\ A_{i,s}= & \sigma_{i|i,s}^{2}{\left(\sigma_{i+1|i,s}^{2}\right)}^{-1} \end{array}$$

In the smoothing algorithm, the initial conditions are $\Delta Z_{i,s}\left(\omega_{s}\right)$ and $\sigma_{I|I,s}^{2}$ for i=I−1,I−2,…,1 and s=1,2,…,S. The covariance smoothing algorithm is used to obtain the covariances between any two states:

(8) $$\sigma_{i,u|I,S}=A_{i,s}\sigma_{i+1,u|I,s}$$

where 1≤i≤u≤I, Equations (7) and (8) are utilized to compute the joint distribution of the increment differences on all of the data. The distribution of any function of the increment differences can be computed by Monte Carlo methods [49,50], and a Monte Carlo estimate of its posterior probability density can be provided by the histogram of the function. The estimate process is empirical Bayes, since it is computed on the basis of maximum likelihood parameter estimates.

### 2.3. Model Parameters and Initial Condition Estimation

In the processing of Kalman filter (5) and (6), Kalman smoother (7), and covariance smoothing (8) algorithms, the initial state variances $\sigma_{0,s}^{2}$, the initial states $\Delta Z_{0,s}\left(\omega_{s}\right)$, and the model parameters $\sigma_{v,s}^{2}$ and $\sigma_{\varepsilon }^{2}$ are assumed to be known; then, an EM algorithm is used to obtain maximum likelihood estimates [46] of the parameters. The details are concluded as follows.

Firstly, the joint probability distribution of $\Delta Z_{1,s}\left(\omega_{s}\right)$ and $\Delta Y_{0,s}\left(\omega_{s}\right)$ at frequency *s* is expressed as:

(9) $$\begin{array}{cc} L_{s} & =p\left(\Delta Z_{0,s}\left(\omega_{s}\right)|\sigma_{v,S}^{2}\right)\times \prod_{i=1}^{I}p\left(\Delta Z_{i,S}\left(\omega_{s}\right)|\Delta Z_{i-1,s}\left(\omega_{s}\right),\sigma_{v,S}^{2}\right)\\ \; & \times \prod_{i=1}^{I}p\left(Y_{i,s}\left(\omega_{s}\right)|\Delta Z_{i,s}\left(\omega_{s}\right),\sigma_{\varepsilon }^{2}\right) \end{array}$$

Assume that the probability density of the initial state is obtained by:

(10) $$p\left(\Delta Z_{0,s}\left(\omega_{s}\right)\right)={\left(\pi \sigma_{v,s}^{2}\right)}^{-1}\exp\left(-\frac{{∥\Delta Z_{0,s}\left(\omega_{s}\right)∥}^{2}}{\sigma_{v,S}^{2}}\right)$$

**E-step**: In iteration it of the E-step, on the basis of the previous estimates of the parameters and observed data from iteration $\left(it-1\right)$, the expectation of the complete data log-likelihood can be calculated. For easy readability, Θ is introduced to represent the parameters $\left\{\sigma_{\varepsilon }^{2},\sigma_{v,S}^{2},\Delta Z_{0,s},\sigma_{0,s}^{2}\right\}$. Analyzing log and expectation to the likelihood yields:

(11) $$\begin{array}{cc} E\left[\log L_{s}^{it}|\cdot Y_{1:I,S},\Theta^{\left(it-1\right)}\right]= & E\left[-\frac{{∥\Delta z_{0,S}^{it}\left(\omega_{s}\right)∥}^{2}}{\sigma_{v,S}^{2,(it-1)}}-\right.\frac{1}{\sigma_{\varepsilon }^{2,(it-1)}}\sum_{i=1}^{I}{∥Y_{i,S}^{it}-\Delta Z_{i,s}^{it}\left(\omega_{s}\right)∥}^{2}\\ \; & -\left(p-1\right)\log\left(\pi \sigma_{\varepsilon }^{2,(it-1)}\right)-\left(p-1\right)\log\left(\pi \sigma_{v,S}^{2,(it-1)}\right)-\\ \; & \left.\frac{1}{\sigma_{v,s}^{2,(it-1)}}\sum_{i=1}^{I}{∥\Delta Z_{i,s}^{it}\left(\omega_{s}\right)-\Delta Z_{i-1,s}^{it}\left(\omega_{s}\right)∥}^{2}|Y_{1:I,s}\right] \end{array}$$

Three quantities are required to be calculated to evaluate Equation (11).

(12) $$\begin{array}{c} \Delta Z_{i|I,S}^{it}\left(\omega_{s}\right)=E\left[\Delta Z_{i,s}\left(\omega_{S}\right)|Y_{1:I,s},\Theta^{\left(it-1\right)}\right]\\ W_{i|I,S}^{it=}=E\left[{∥\Delta Z_{i,s}\left(\omega_{s}\right)∥}^{2}|Y_{1:I,S},\Theta^{\left(it-1\right)}\right]\\ W_{i,i-1|I,s}^{it=}=E\left[\Delta Z_{i,s}\left(\omega_{s}\right)Z_{i-1,s}^{*}|Y_{1:I,s},\Theta^{\left(it-1\right)}\right] \end{array}$$

By using the Kalman filter (5) and (6), Kalman smoothing (7), and covariance smoothing algorithms (8), these three quantities can be efficiently computed.

**M-step**: Let ${\tau_{\left(v,s\right)}^{it}=1\;/\sigma }_{v,s}^{2,it}$ and ${\tau_{\varepsilon }^{it}=1/\sigma }_{\varepsilon }^{2,it}$, then each gamma prior density is defined as:

(13) $$e\rho \left(\tau |\alpha ,\beta \right)=\frac{\beta^{\alpha }}{\Gamma \left(\alpha \right)}{\left(\tau \right)}^{\alpha -1}\exp\left(-\beta \tau \right)$$

For α>1 and β>0, the expectation of log joint posterior density of each parameter is:

(14) $$\begin{array}{cc} \; & E\left[\log ep\left(\tau_{\varepsilon }^{it},\tau_{v,s}^{it}\right)Y_{1:p-1,s},\Theta^{it-1}\right]\propto \\ \; & \log\left(e\rho \left(\tau_{\varepsilon }^{it}|\alpha ,\beta \right)\right)+\log\left(e\rho \left(\tau_{k,s}^{it}|\alpha ,\beta \right)\right)+E\left[\log L_{s}^{it}\right] \end{array}$$

Equation (14) is required to be maximized with respect to $\tau_{v,s}^{it}$ and $\tau_{\varepsilon }^{it}$, yielding the results:

(15) $$\begin{array}{c} \tau_{v,s}^{it}=\frac{p-1+\alpha }{2\sum_{i=1}^{it}\left(W_{i-1|I,s}^{it}-R\left\{W_{i,i-1|I,s}^{it}\right\}\right)+W_{I|I,s}^{it}+\beta }\\ \tau_{\varepsilon }^{it}=\frac{\alpha -1+S(p-1)}{\sum_{i,s}\left({\left(Y_{i,s}^{it}\right)}^{2}+W_{i|I,S}^{it}-2R\left\{Y_{i,s}^{it}*\Delta Z_{i|I,S}^{it}\right\}\right)+\beta } \end{array}$$

In addition, each initial state $\Delta Z_{0,s}$ and initial variance $\sigma_{0,s}^{2}$ can be computed as:

(16) $$\begin{array}{cc} \Delta & Z_{s}\left(\omega_{s}\right)=Y_{1}*SCT_{s}\\ \sigma_{s}^{2} & =\Delta Z_{s}\circ {\left(\Delta Z_{s}\right)}^{*} \end{array}$$

where ∘ denotes the Hadamard product, the EM algorithm iterates between **E-steps** and **M-steps** until satisfying Equation (17) or ${it=EM}_{max}$, and ${EM}_{max}$ is a predefined number of maximum iterations and $\epsilon \in \left(0,0.001\right)$.

(17) $$\frac{{∥\Delta Z_{i|I,S}^{it}-\Delta Z_{i|I,S}^{it-1}∥}^{2}}{\Delta Z_{i|I,s}^{it-1}}<\epsilon$$

The details are described in Algorithm 1.
**Algorithm 1** BE-SCTEquations**Input:** matrix $Y_{i,s}$, maximum iteration of EM steps ${EM}_{max}$, minor integer ϵ.    **while** $it<{EM}_{max}$ and $\epsilon_{it}<\epsilon$ **do**          Generating initial states $\Delta Z_{0,s}$ and initial variances $\sigma_{0,s}^{2}$ by Equation (16);          **for** i=2,…,I **do**               Obtaining quantities in Equation (12) by recursively solving Equations (5) and (6)     to evaluate Equation (11).          **end for**          **for** i=I−1,…,1 **do**                Recursively computing Equations (7) and (8), to get quantities in Equations (11)     and (12).          **end for**          Obtaining final $\tau_{v,s}^{it}$ and $\tau_{\varepsilon }^{it}$ by Equation (15);          it=it+1.          **for** i=2,…,I **do**                On the basis of estimated parameters, recursively solving Equations (5) and (6) to    perform the Kalman filter in matrix $Y_{i,s}$.          **end for**    **end while****Output:** Final matrix $Y_{i,s}^{\left(BE-SCT\right)}$ with *s* lines and *i* columns.

### 2.4. Numerical Experiments

In this section, two numerical simulations are used to demonstrate the effectiveness of the proposed BE-SCT. To add an extra degree of difficulty in analysis, additive Gaussian noise with a standard deviation of 0.1 and a mean of zero is artificially induced to the nonstationary nonlinear analytical signal. The sampling frequency is set to 100 Hz, and the time-frequency representation obtained by the proposed BE-SCT is compared with the continuous WT (CWT), HHT, and SCT.

The first example is given by:

(18) $$f\left(t\right)=\left\{\begin{array}{cc} \sin\left(2\pi \left(25t+10\sin t\right)\right) & \left(0\leq t\leq 6\;s\right)\\ \sin\left(2\pi \left(34.2t\right)\right) & \left(6\;s\leq t\leq 10\;s\right) \end{array}\right.$$

**Figure 1:**
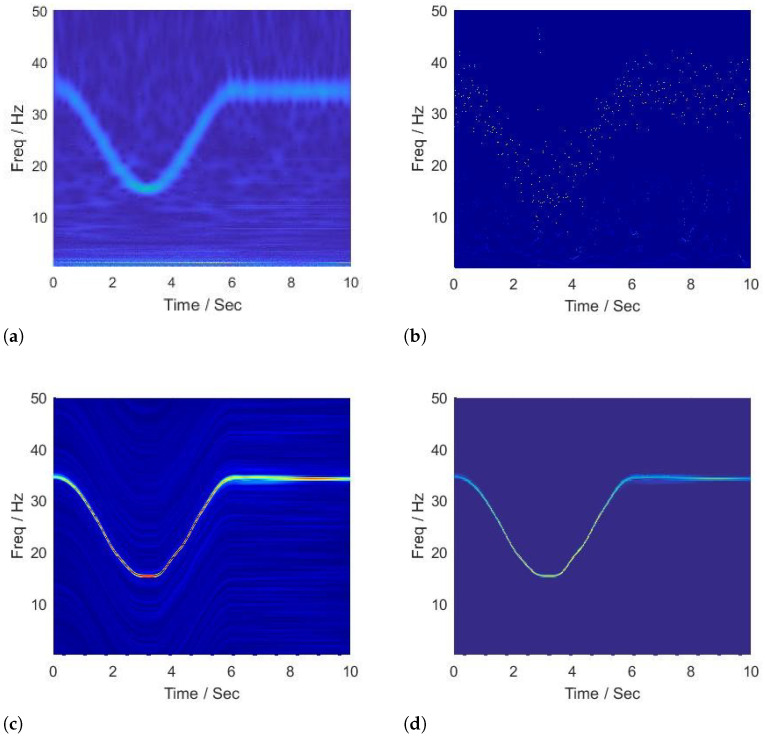
Time-frequency analysis of signal with instantaneous frequency in Equation (19) by (**a**) CWT, (**b**) HHT, (**c**) SCT, and (**d**) BE-SCT.

The SNR of the first signal is 7.1203 dB, and the time-frequency representations generated by the CWT, HHT, SCT, and BE-SCT are shown in Figure 1. The wavelet is set to be ‘cmor3-3’, and the total scale is 256, the representation given by the CWT, as shown in Figure 1a. It can be seen that the CWT dissipates the energy around the instantaneous frequency at the high-frequency plane because of its coarse frequency resolution. Moreover, the representation is too blurry to reveal the time-frequency trajectory due to the high sensitivity to corruptions. In Figure 1b, HHT has higher anti-noise and robust performance compared with CWT; however, due to the misleading energy frequency distribution for corruptions and intrinsic modal information, the analytical consequence is too sparse to reveal the instantaneous frequency trajectory. As shown in Figure 1c, SCT provides excellent local estimates of signal features, but on account of its inability to offer a statistical inference framework appropriate for the entire time series, multiple instantaneous frequency trajectories are generated in the time-frequency plane. Figure 1d shows that the BE-SCT outperforms the CWT, the HHT, and the SCT as it clearly reveals the true time-frequency pattern of the analytical signal.

The second example is given by:

(19) $$f\left(t\right)=\sin\left(30+50t+60t^{2}+40\sin t\right)\;\left(0\leq t\leq 10\;s\right)$$

**Figure 2:**
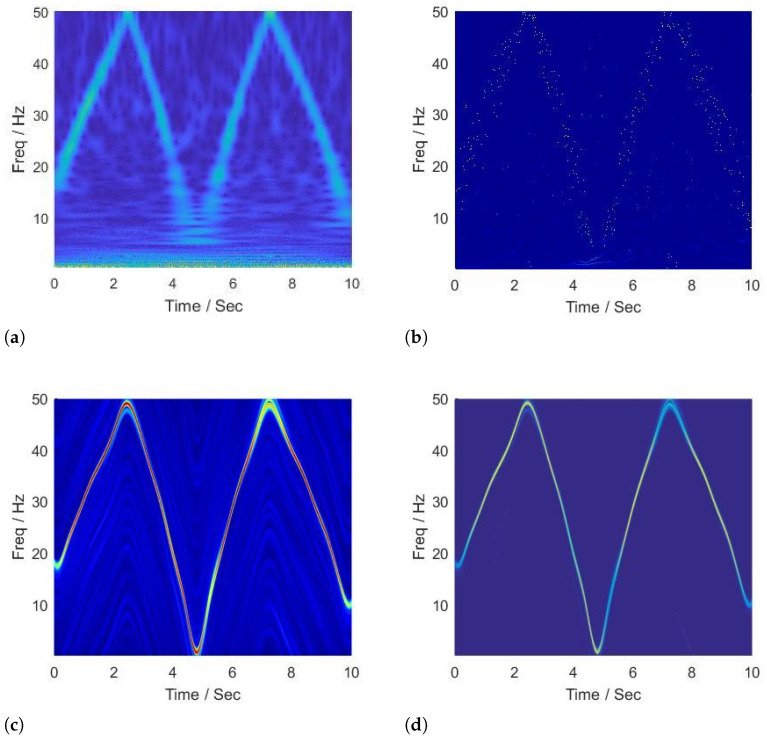
Time-frequency analysis of signal with instantaneous frequency in Equation (19) by (**a**) CWT, (**b**) HHT, (**c**) SCT, and (**d**) BE-SCT.

The SNR of the second signal is 7.1177 dB, and the time-frequency representations generated by the CWT, HHT, SCT, and BE-SCT are shown in Figure 2. As shown in Figure 2a, CWT shows poor resolution in the time-frequency plane; besides, due to the reciprocal relationship between the center frequency of the wavelet function and window length, it is difficult for the representation given by the CWT to differentiate the true instantaneous frequency trajectory from the spurious frequency components introduced by the additive corruptions. The representation generated by the HHT is shown in Figure 2b, in which partial spurious frequency contents generated by nonstationary noise have been removed by EMD. However, some intrinsic modal information is mistaken for artifacts, so the true frequency trajectory is still hard to be identified by HHT. As shown in Figure 2c, the SCT offers the instantaneous frequency representation with an excellent concentration, and the most prominent trajectory can characterize the true time-varying frequency successfully. However, the spectrum estimates on adjacent spline functions are not formally related, resulting in the parallel spurious trajectories. On the other hand, as shown in Figure 2d, it is evident that the BE-SCT outperforms the CWT, the HHT, and the SCT; based on the precise parameters estimation, the adjacent spline functions are recursively linked, therefore giving its best performance in this high signal-to-noise spectrogram estimation problem.

## 3. Spectrum-Aware Video Magnification

On the basis of BE-SCT, in this section, a spectrum-aware video magnification technique is presented to amplify small motions within large ones. Our technique has three main components:

1. On the basis of the earth mover’s distance (EMOD) algorithm (readers interested in this theory, please refer to [22]), which avoids quantization and other binning problems, the moment function of original video motion information is temporally extracted;
2. By applying BE-SCT, the estimation stage seeks to understand the time-frequency characteristic of global nonstationary motions in analytical video;
3. With the appropriate prior knowledge, the dynamic ideal band-pass filter is used to remove large motions while preserving subtle ones.

**Figure 3:**
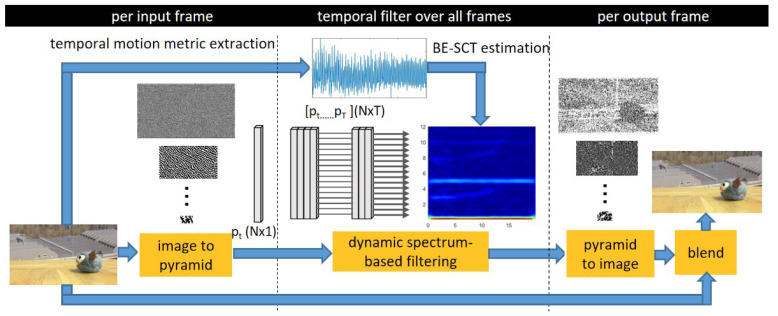
Video spectrum-aware magnification pipeline. Our approach does not require manual region annotation nor additional depth information as done in conventional techniques; instead, by employing the proposed BE-SCT, the intrinsic frequency characteristics can be understood to achieve the goal of adaptive large motions isolation, meanwhile avoiding the nonlinear limitation in the Eulerian acceleration approach.

Our proposed magnification pipeline is depicted in Figure 3.

### 3.1. Motion Metric Extraction

Carrying out the analysis of global motion information in video remains a challenging task due to the millions of pixels’ respective temporal vibration signal exiting in video. In engineering application areas and stereo-vision systems [51,52,53,54,55,56,57], the three-dimensional digital image correlation (3D DIC) and three-dimensional point tracking (3DPT) techniques are used to extract the full-field dynamic displacements of the analytical structure, and temporal vibration signals are further analyzed to obtain the material properties [58,59]. These methods are appropriate for structural engineering due to the law of mechanics; however, limitations arise when analyzing an irregular video. The selection of degrees of freedom is time consuming and error prone. Inspired by recent research [60], which extracts the periodic pulsation of flame from the temporal image sequence based on the Euclidean distance and cross-correlation coefficient, EMOD is a method measuring a distance between two distributions. In this paper, every video frame is considered as a distribution, and EMOD is used to calculate the distance between each frame and the first frame. Therefore, temporal EMOD metrics can be conducted; then, BE-SCT is applied to these temporal metrics to generate the time-frequency estimation in the following parts. Detailed information of the EMOD can be seen in the reference [61].

### 3.2. Dynamic Spectrum-Aware Filtering

Applying the specially constructed BE-SCT, which has been verified for the superiority over other time-frequency estimation algorithms, the true time-varying frequency pattern of global motions in analytical video can be precisely obtained. Hereby, it can be observed that at the scale of subtle visual changes, the frequency of large motion is relatively small. By only magnifying small deviations of dynamically selected frequency ranges, this method arrives at temporal spectrum-aware filtering magnification.

For easy readability of the temporal spectrum-aware filtering, a time-domain mathematics model is established for illustration. Consider the time domain of intensity changes denoted by $I\left(x,y,t\right)$ at position $\left(x,y\right)$ and time *t* based on the significant anti-noise performance of complex steerable pyramid, small temporal variations in the spatial offset of edges can be converted to subtle temporal changes in polar coordinates of the complex filter responses in the pyramid. Therefore, in the temporal mathematics model, the temporal variations are first turned into the frequency domain by Fourier transform, which is $S\left(\omega ,\rho ,t\right)$. Then, based on the observation that the large motions differ evidently from small ones in frequency property, the sophisticated time domain of intensity variations is reconstructed in the frequency domain as a combination of two components:

(20) $$S\left(\omega ,\rho ,t\right)=S_{\rho a}\left(\omega ,\rho ,t\right)+S_{\rho b}\left(\omega ,\rho ,t\right)$$

where $S_{\rho a}\left(\omega ,\rho ,t\right)$ denotes the variations component with spectral amplitude above the threshold ρ, and $S_{\rho b}\left(\omega ,\rho ,t\right)$ stands for the opposite.

Ultimately, it is crucial to develop a self-adapting estimation for the spectrum-aware filtering to separate these two components. Returning to the proposed BE-SCT, it can act not only as the “detector” of the property of global video motions but also adaptively isolate the large motions from the small ones as deeper utilized. On the basis of the time-varying spectrogram by BE-SCT, this method further constructs a dynamic frequency-based filtering algorithm to handle the challenging isolation task. In the actual operation, similar to the common ideal band-pass filter, the time-domain dynamic weighting function of the spectrum-aware filtering is defined as:

(21) $$W_{\omega ,\rho ,t}=\left\{\begin{array}{c} 1,\;\rho \in \left[\rho_{l},\rho_{h}\right]\\ 0,\;\mathrm{otherwise} \end{array}\right.$$

where $\left[\rho_{l},\rho_{h}\right]$ stands for the identified amplitude-frequency ranges in the time-domain spectrogram generated by BE-SCT. $\rho_{l}$ is the minimum amplitude bound, which is not critical, since small noise can be negligible after arbitrary simple built-in spatial filtering algorithms. $\rho_{h}$ is the maximum amplitude threshold used for eliminating the large motions, which can be experience-modifiable.

## 4. Experimental Results

To verify the effectiveness of the proposed spectrum-aware approach, experiments on real sequences as well as on a synthetically generated one with ground truth are performed. This paper only assesses the real videos’ performance qualitatively, whereas for the synthetic sequence, the quantitative evaluation is taken against ground truth. For all videos, the video frames are processed in a YIQ color space. In the contrast tests, a complex steerable pyramid with octave bandwidth filters and four orientations is used to decompose each frame into phase and magnitude. The results demonstrate that state-of-the-art techniques optimized for subtle variations generate blurs and artifacts when handling large motions. Our technique fully utilizes the powerful BE-SCT, significantly reduces haloes or corruptions, and increases the scope of its applicability.

### 4.1. Real-Life Sequences

**Figure 4:**
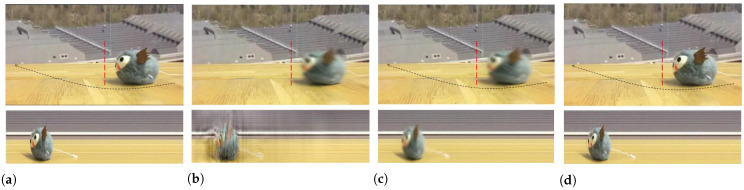
A cat toy vibrating at a high frequency, along with the large amplitude movement of a circle trajectory depicted by the black arrow. Four frames indicating the toy’s trajectory are shown in each top row, the bottom rows show the spatio-temporal line corresponding to the green line in the relevant video frames. (**a**) Original video. (**b**) Phase-based video magnification. (**c**) Eulerian-acceleration magnification. (**d**) Our proposed spectrum-aware magnification. The proposed magnification approach can clearly reveal the vibration of the cat toy without inducing blurs and artifacts [21].

Figure 4 shows a cat toy moving on the table, which coincided with the high-frequency vibration perpendicular to the circle trajectory. The goal of this experiment is to magnify the vibration with amplification factor α=8; the motion above the black arrow is recorded in the spatio-temporal slice indicated with the green line over the raw video. The phase-based motion magnification generates substantial artifacts due to the large movement on the table. The Eulerian-acceleration approach relies on the second-order filter; therefore, the nonlinear motions in the background are magnified while inducing tiny blurring effects, as seen in the figure. Our proposed technique manages to achieve this by amplifying the variations at the pixels that lie in the time-varing frequency property estimated by BE-SCT, thus magnifying the vibration of the toy and separating the motion along the trajectory on the table.

**Figure 5:**
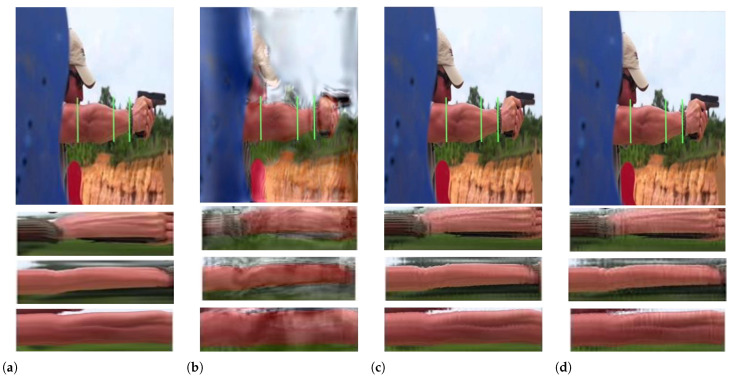
In the gun shooting sequence, the strong recoil causes small vibrations of the arm. The spatio-temporal slice is shown at different positions with three green lines over the sequence for each processing. (**a**) Original video frame. (**b**) Phase-based video magnification. (**c**) Eulerian-acceleration magnification. (**d**) Our proposed spectrum-aware magnification. The Eulerian-acceleration approach only magnifies the nonlinear motion by linking the response of a second-order Gaussian derivative, whereas the phase-based method results in large blurs and artifacts. Our proposed method magnifies the arm movements correctly without being affected by the background clutter [21].

Figure 5 demonstrates various motion amplification results for a gun shooting video with magnification factor α=8. In this case, the recoil of the gun induces subtle movement in the arm muscles. To preform an in-depth and meticulous analysis, the movements of the bracelet, upper limb, and the forearm are recorded in the spatio-temporal slices indicated with three green lines over the original sequence. Due to the strong arm movement, the phase-based processing induces ripples and motion artifacts, which cover the subtle motion in the muscles. The Eulerian-acceleration method only magnifies the nonlinear motion, leading to the loss of linear subtle movement. Our proposed technique not only magnifies the intensity changes of the arm muscles but also magnifies clearly the intensity variations of the bracelet, which is caused by the reflection of the muscles, as shown in the plot on the bottom-right of Figure 5.

**Figure 6:**
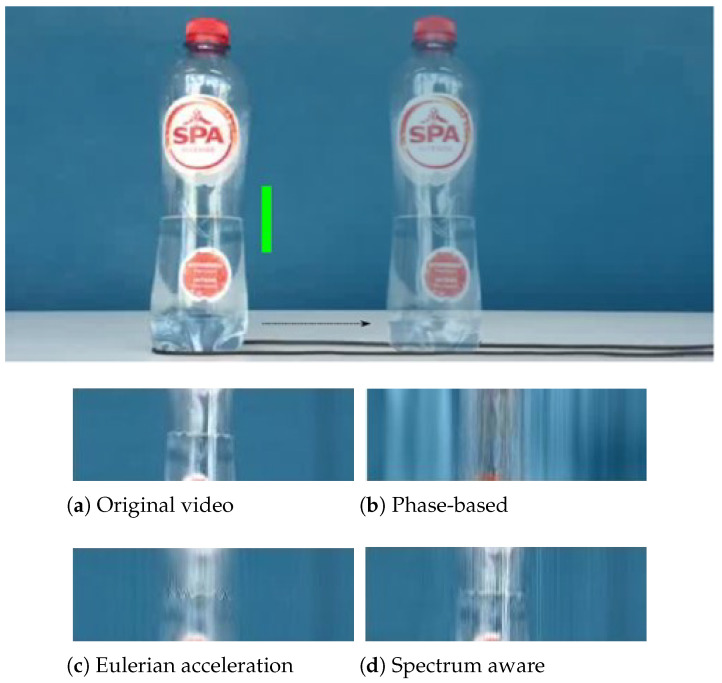
The water oscillating in a bottle while the bottle is being pulled sideways on a smooth surface. The green stripe indicates the locations at which the dynamic movements are temporally detected from the video. Compared to the state-of-the-art approaches, our proposed magnification method is able to amplify the oscillations in the water while not inducing substantial blurs [21].

In Figure 6, the picture shows a transparent bottle with water being pulled sideways on the smooth surface, whereas the level of water in the bottle fluctuates sharply, as shown in the original sequence. The magnification factor α=8 is chosen for each video processing. According to the contrast experiments, the phase-based approach generates significant blurring artifacts caused by the bottle moving. On the other hand, similar but more precise than Eulerian-acceleration processing, our approach is able to correctly amplify the desired motion—oscillation of the water level, while not inducing substantial blurring artifacts.

**Figure 7:**
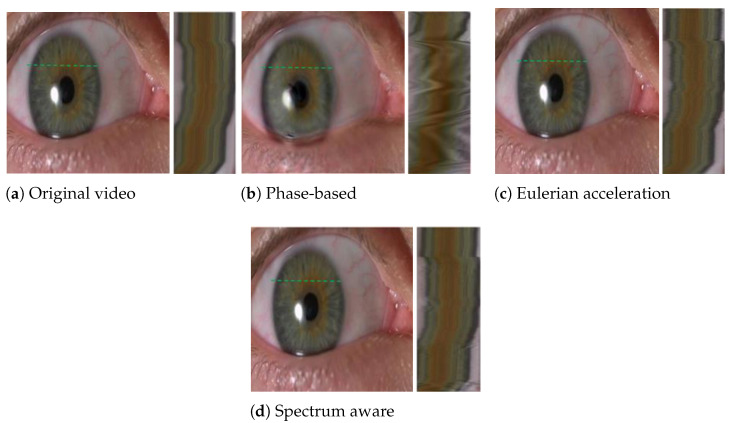
The eye video and its magnification with the phase-based approach, the Eulerian-acceleration approach, and our spectrum-aware processing. The spatio-temporal slice is shown in each approach for the green stripe (top-left). This video demonstrates an eye moving along the horizontal direction, as shown in the original sequence; such wobbling is too subtle to be observed (top-left). The global motion of the eye generates significant blurring artifacts when processed with the phase-based approach. However, processing the sequence with Eulerian acceleration and our approach show clearly that the iris wobbles as the eye moves; through the in-depth comparison, more local details can be preserved in our approach [19].

Figure 7 shows magnification results for iris wobbling, combined with large-scale eye horizontal movements, and sets the magnification factor α=15. As demonstrated in the figure (top-right), when applied to the video with the phase-based technique, the small motion remains hard to be seen because it is overshadowed by the then-magnified large motions and its blurring artifacts. Our spectrum-aware magnification maintains the local motions of the iris wobbling. Eulerian acceleration does magnify segmental temporal variations; however, it kills more useful information than our approach.

**Figure 8:**
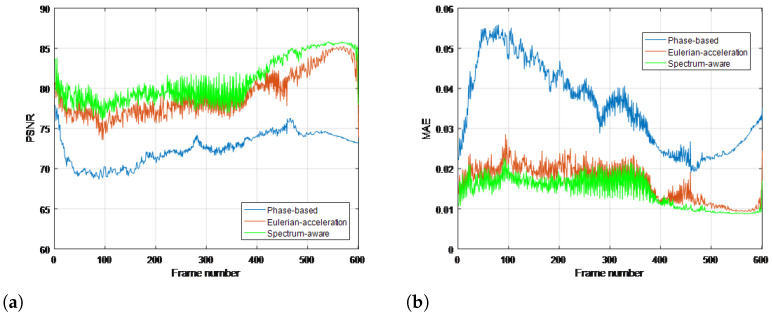
Objective metrics with ground truth for each magnification processing using (**a**) PSNR and (**b**) MAE in cat toy video.

**Figure 9:**
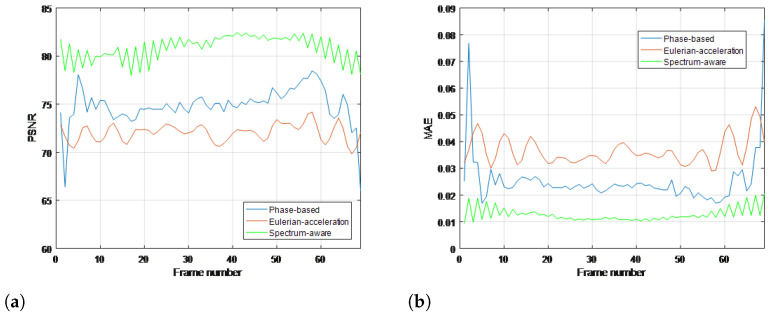
Objective metrics with ground truth for each magnification processing using (**a**) PSNR and (**b**) MAE in gun shooting video.

**Figure 10:**
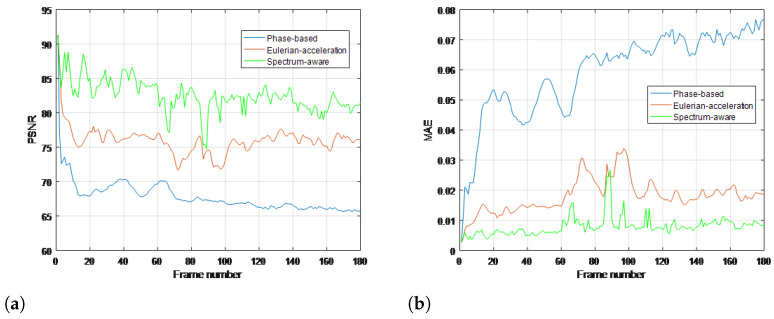
Objective metrics with ground truth for each magnification processing using (**a**) PSNR and (**b**) MAE in bottle video.

**Figure 11:**
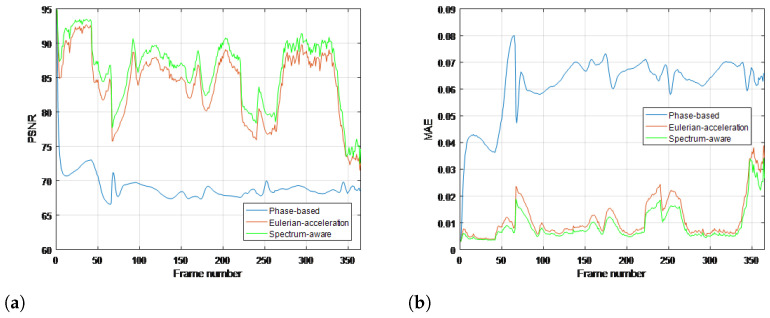
Objective metrics with ground truth for each magnification processing using (**a**) PSNR and (**b**) MAE in eye video.

To quantitatively evaluate the performance of the proposed method compared with traditional methods, commonly used objective metrics including peak signal-to-noise ratio (PSNR) and mean absolute error (MAE) are further introduced, which are measured over the whole image in all frames. The range of MAE at the numerical span is [0, 1]; the closer to 0, the more similarities there are in the two images. Meanwhile, PSNR follows the opposite monotonicity rule. Results for all the real-life videos are given in Figure 8, Figure 9, Figure 10 and Figure 11, respectively. It can be seen that the proposed method achieves higher values of PSNR and lower values of MAE than the traditional methods, quantitatively validating its superiority in terms of magnifying subtle changes and achieving the best anti-noise results.

### 4.2. Synthetic Sequence

**Figure 12:**
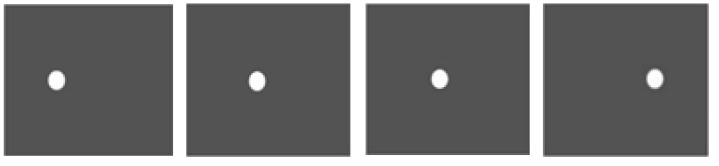
Synthetic video. A ball moves from left to right along with a tiny vibration in the vertical direction [21].

In Figure 12, the picture demonstrates a synthetic ball that moves horizontally on the screen from the left to right corner; the radius of the ball is set to 10 pixels, and the velocity of movement is 1 pixel/frame. The ball vibrations are modeled in the vertical direction as a sine wave, with a maximum value of 1 pixel. The vibration frequency is 3 cycle/sec, and the frame rate is set to 30 frame/sec. For ground truth amplification, the temporal changes are magnified by two times without changing any other parameters. A complex steerable pyramid is applied for all contrast processes, with octave bandwidth filters and four orientations, which only amplify the pyramid level with a magnification factor of 5.

**Figure 13:**
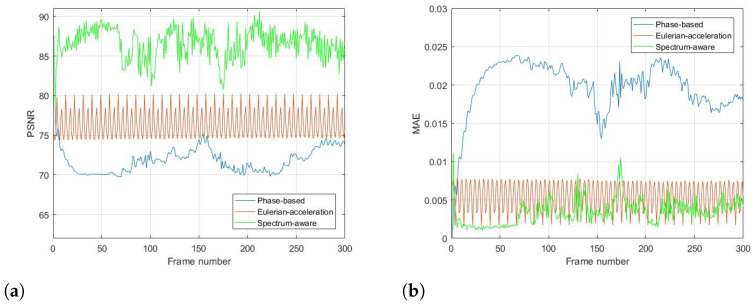
Objective metrics with ground truth for each magnification processing using (**a**) PSNR and (**b**) MAE in synthetic video.

Objective results are given in Figure 13. Statistically speaking, our proposed algorithm yields the best performance for deriving the most significant fidelity. However, on the other hand, due to the innovative construction of second-order Gaussian derivative, the magnified sequence processed by the Eulerian-acceleration approach shows certain regularity in the time domain, which provides us some beneficial enlightenment of fundamental improvements for the future work.

## 5. Discussion

**Limitation of our approach.** As demonstrated in the controlled experiments for synthetic sequence magnification, compared with the Eulerian-acceleration approach, our magnified results show relatively irregular temporal variations. However, our algorithm achieves the best anti-noise performance and retains the most details over other methods, as verified in each controlled experiment. If required to recover regular small motions from large ones as possible in some special project applications, the Eulerian-acceleration approach may be the best choice in spite of its other flaws. Thus, to establish a more comprehensive analytical framework, some powerful shift rules will be constructed in the pyramid level in future research.

**Performance superiority over state of the art.** The superiority of our work can not only be summarized in the context of time-frequency analysis but also in video motion further revelation. For the time-varying spectral analysis of nonstationary nonlinear signals, this paper introduces the SCT, which has been validated for its excellent local estimates of data features. A statistical inference framework is established to efficiently relate its spectral estimates across local intervals; the important advance that this paper presents is BE-SCT. In Figure 1 and Figure 2, BE-SCT depicts clearly the time-varying spectrum trajectory of two nonstationary nonlinear signals, whereas the state-of-the-art approaches do not. For large movements isolation, this method sidesteps the problem of Eulerian-acceleration processing for being easily affected by the nonlinear background clutter. The results in Figure 4, Figure 5, Figure 6, Figure 7, Figure 8, Figure 9, Figure 10, Figure 11, Figure 12 and Figure 13 for processing whether real-life or synthetic sequences show the robustness superiority of our spectrum-aware technique. Therefore, in the future work, further extensions will be presented of its anti-noise ability.

## 6. Conclusions

Standard video magnification techniques cannot reliably handle large motions, which are bounded by excessive user annotations, additional depth information, their inability to operate nonlinear background clutter, and so on. By exploiting the spectrum characteristic of global motions in analytical video, we are not restricted by such limitations and can magnify unconstrained videos.

To construct a powerful time-varying spectral analytical framework, the spectral representation theorem-based inference model is adapted to SCT. Then, with the assistance of EMOD extraction, background large movement values are ignored by filter responses at spectrum layers.

Spectrum-aware motion magnification is demonstrated on several real-world and synthetic sequences. We show that our approach performs well, has better anti-noise characteristics, and has less background edge artifact than the state of the art. Improving robustness in the pyramid level so that it works at higher magnification is an important direction for future work.
